# Conditional Expression of TGF-β1 in Skeletal Muscles Causes Endomysial Fibrosis and Myofibers Atrophy

**DOI:** 10.1371/journal.pone.0079356

**Published:** 2013-11-14

**Authors:** Jigna Narola, Sachchida Nand Pandey, Adam Glick, Yi-Wen Chen

**Affiliations:** 1 Research Center for Genetic Medicine, Children’s National Medical Center, Washington, DC, United States of America; 2 Department of Veterinary and Biomedical Sciences, Pennsylvania State University, University Park, Pennsylvania, United States of America; 3 Department of Integrative Systems Biology and Department of Pediatrics, George Washington University, Washington, DC, United States of America; Sanjay Gandhi Medical Institute, India

## Abstract

To study the effects of transforming growth factor beta 1 (TGF-β1) on fibrosis and failure of regeneration of skeletal muscles, we generated a tet-repressible muscle-specific *TGF-β1* transgenic mouse in which expression of TGF-β1 is controlled by oral doxycycline. The mice developed muscle weakness and atrophy after TGF-β1 over-expression. We defined the group of mice that showed phenotype within 2 weeks as early onset (EO) and the rest as late onset (LO), which allowed us to further examine phenotypic differences between the groups. While only mice in the EO group showed significant muscle weakness, pathological changes including endomysial fibrosis and smaller myofibers were observed in both groups at two weeks after the TGF-β1 was over-expressed. In addition, the size of the myofibers and collagen accumulation were significantly different between the two groups. The amount of latent and active TGF-β1 in the muscle and circulation were significantly higher in the EO group compared to the LO or control groups. The up-regulation of the latent TGF-β1 indicated that endogenous TGF-β1 was induced by the expression of the TGF-β1 transgene. Our studies showed that the primary effects of TGF-β1 over-expression in skeletal muscles are muscle wasting and endomysial fibrosis. In addition, the severity of the pathology is associated with the total amount of TGF-β1 and the expression of endogenous TGF-β1. The findings suggest that an auto-feedback loop of TGF-β1 may contribute to the severity of phenotypes.

## Introduction

Transforming growth factor beta 1 (TGF-β1) belongs to a family of multifunctional cytokines including bone morphogenic proteins (BMPs) and activins [1]. TGF-β1 plays essential roles in various biological processes, including cell growth, differentiation, apoptosis, tissue development, and inflammation [1], [2], [3]. TGF-β1 stimulates synthesis of extracellular matrix (ECM) proteins and inhibits matrix degradation, resulting in the promotion of fibrosis and tissue repair. This phenomenon has been proposed to play a central role in fibrotic tissue development in certain lung, muscle, hypertensive vascular, and diabetic renal diseases [4], [5], [6], [7], [8], [9], [10]. Besides its fibrogenic effects, studies showed that TGF-β1 is also a potent inhibitor of growth and differentiation of myoblasts and vascular smooth muscle cells (VSMCs) [11], [12], [13], and can suppress division and block fusion of satellite cells both *in vitro* and *in vivo* through suppressing myogenic factors [11], [12], [14], [15], [16], [17], [18].

After TGF-β1 is translated, it is cleaved by intracellular proteolytic processes. The N-terminus of the propeptide, also known as latency associated peptide (LAP), keeps the TGF-β1 latent. The TGF-β1 is secreted as part of an inactive tripartite complex consisting of a homodimer of the TGF-β1 with LAP, and a molecule of latent TGF binding protein (LTBP) [19]. The complex is transported to the ECM and is the major form of TGF-β1 found *in vivo* [19], [20]. LTBPs interact with various matrix components including collagen and fibronectin [21]. TGF-β1 must be released from this complex before it can interact with TGF-β receptors. During latent TGF-β1 activation, the TGF-β1 is released from LAP.

TGF-β1 is up-regulated in muscles of Duchene muscular dystrophy (DMD), Congenital muscular dystrophy, and Inflammatory myositis [5], [22], and *TGF-β* mRNA expression was correlated with the severity of fibrosis in dystrophic muscles [5]. It was reported to be localized in myofibers and perimysial connective tissue. Our previous temporal expression profiling study showed that genes involved in TGF-β pathway were up-regulated at the symptomatic stage of DMD but not differentially expressed at the asymptomatic stage, suggesting that the activation of TGF-β1 pathways was secondary to the primary dystrophin deficiency and may play a critical role in the fibrosis and failure of muscle regeneration in DMD [23]. In addition, suppressing the TGF-β1 pathway in *mdx* mice, a mouse model of DMD, has been shown to improve disease phenotypes [24], [25]. To clearly define the effect of TGF-β1 over-expression in healthy skeletal muscles, we generated and characterized a tet-repressible muscle-specific transgenic *TGF-β1* mouse in which the over-expression of TGF-β1 is restricted in skeletal muscles and can be controlled by oral administration of doxycycline. The findings will help us determine the contribution of TGF-β1 to the pathologies including fibrosis and failure of regeneration seen in muscle disorders.

## Results

**Figure 1:**
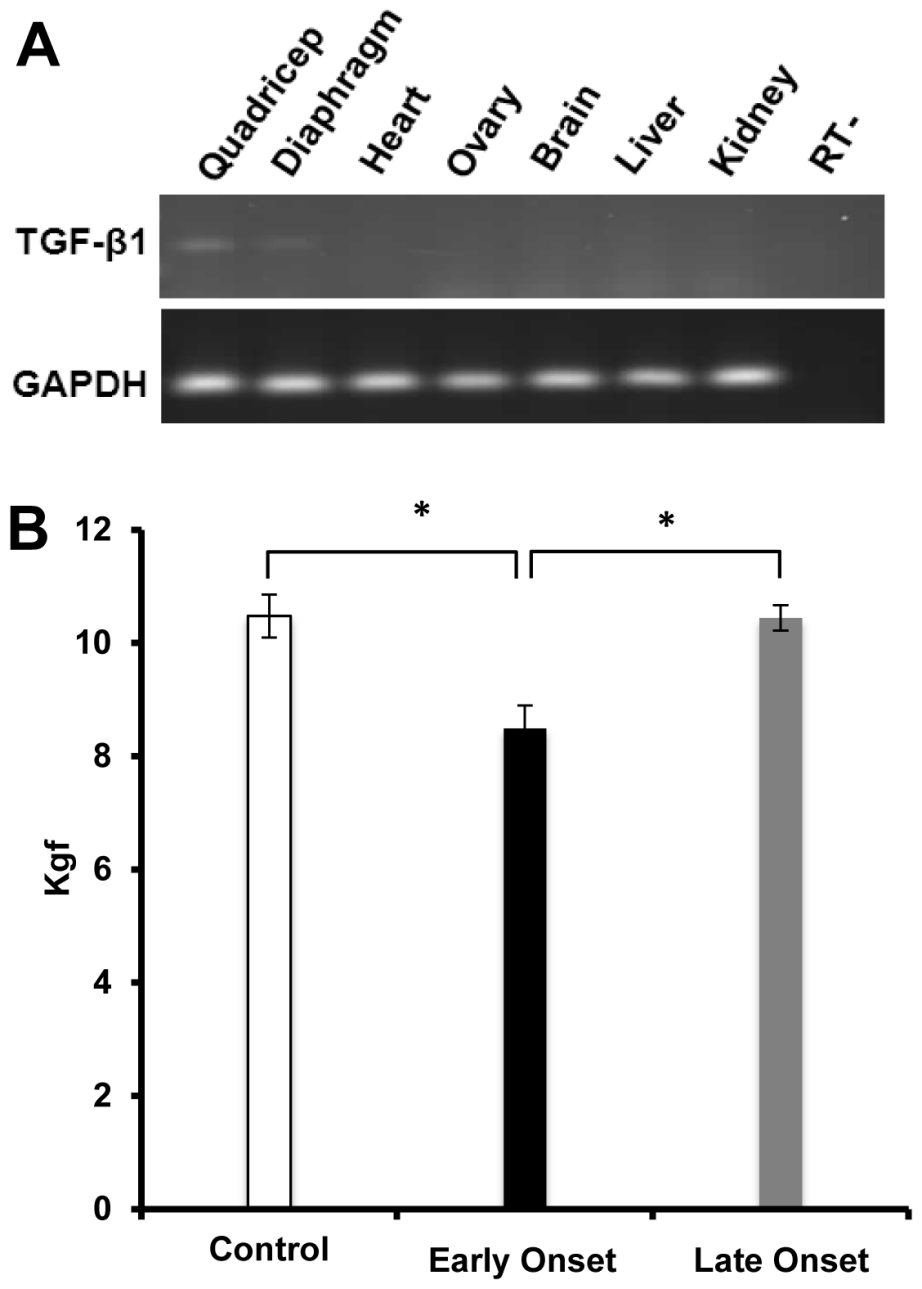
Muscle weakness caused by TGF-β1 over-expression in skeletal muscles after doxycycline removal. (A) Expression of the *TGF-β1* mRNA in various organs. *TGF-β1* transcripts were detected in the two muscles, quadriceps and diaphragm, examined (lanes 1 and 2), but not in the brain, heart, lung, liver, kidney or ovary (lanes 3–7, respectively). Lane 8 is a no RT control. (B) GSM showed that muscle strength was significantly reduced in mice that developed early phenotype but not the rest of the mice over-expressing TGF-β1. The asterisks indicate significant differences with *p*<0.05.

To confirm muscle-specific expression, we first detected the expression of *TGF-β1* transgene in the *mCK-tTA/TRE-TGF-β1* mice by RT-PCR. *TGF-β1* mRNA was detected in muscles, including quadriceps and diaphragm after the transgene was induced for 2 weeks. Transgene expression was not detected in other organs examined including heart, brain, kidney, liver, ovaries, and lung (Figure 1A).

To characterize the phenotype of the *mCK-tTA/TRE-TGF-β1* mice, we first induced *TGF-β1* transgene expression by discontinuing oral doxycycline, followed by 15 weeks of observation. The mice were considered having a disease phenotype when they showed body weight loss in combination with muscle weakness by grip strength. We observed variability in onset of disease phenotype in *mCK-tTA/TRE-TGF-β1* mice after TGF-β1 was induced in the muscles. Among the 20 mice studied, 40% of the mice showed body weight loss and muscles weakness within 2 weeks. Approximately 30% of the mice showed the phenotype between 5 to12 weeks after the transgene induction, and 30% of the *mCK-tTA/TRE-TGF-β1* mice did not exhibit any phenotype during the observation period of 15 weeks. To further characterize the mouse phenotype, we defined the group of mice that showed phenotype within 2 weeks as early onset (EO) and the rest of mice as late onset (LO). This allows us to further examine the differences in muscle strength and pathology in these mice.

### The *mCK-tTA/TRE-TGF-β1* Mice Developed Muscle Weakness and Myofibers Atrophy after the *TGF-β1* Transgene was Induced

**Figure 2:**
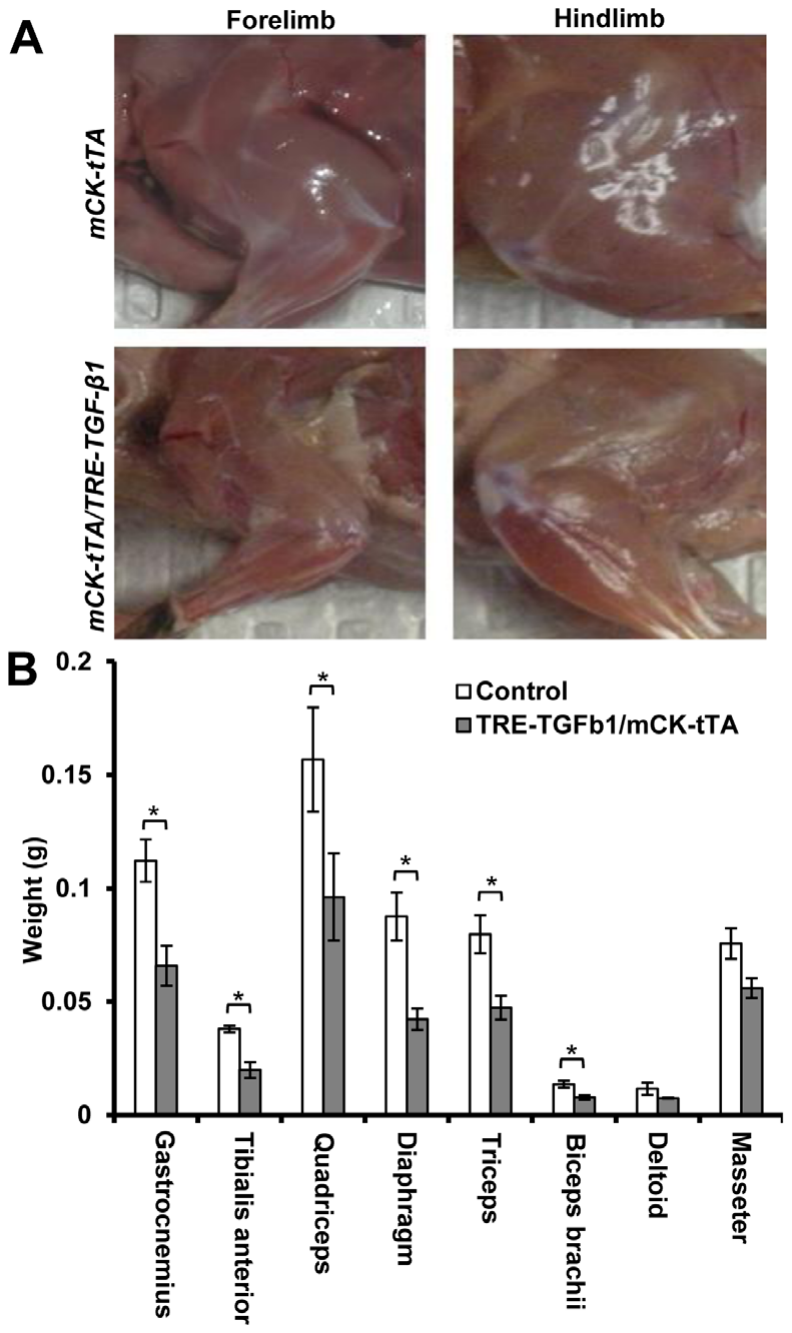
Skeletal muscle wasting due to TGF-β1 over-expression. (A) Skeletal muscle wasting in mice over-expressing TGF-β1, which developed muscle weakness. (B) The average muscle weight (g, mean ± s.e.m.) was reduced in these mice. “*” indicates p<0.05 and “**” indicates p<0.01.

Muscle function of the EO, LO and control mice were examined one week after TGF-β1 was induced using a grip strength assay. We observed that the hindlimb muscle strength of the mice in the EO group was reduced by 11.2% (p<0.05) in comparison with the control mice (Figure 1B). At this time point grip strength of the mice in the LO group was not significantly different from the control mice. For the mice that showed muscle weakness, visible muscle atrophy of both forelimbs and hindlimbs was observed (Figure 2A). The weight of gastrocnemius, tibialis anterior, quadriceps, biceps brachii, diaphragm and triceps muscles were significantly reduced (p<0.05) in these mice while the changes of deltoid and masseter muscle were not statistically significant (Figure 2B).

**Figure 3:**
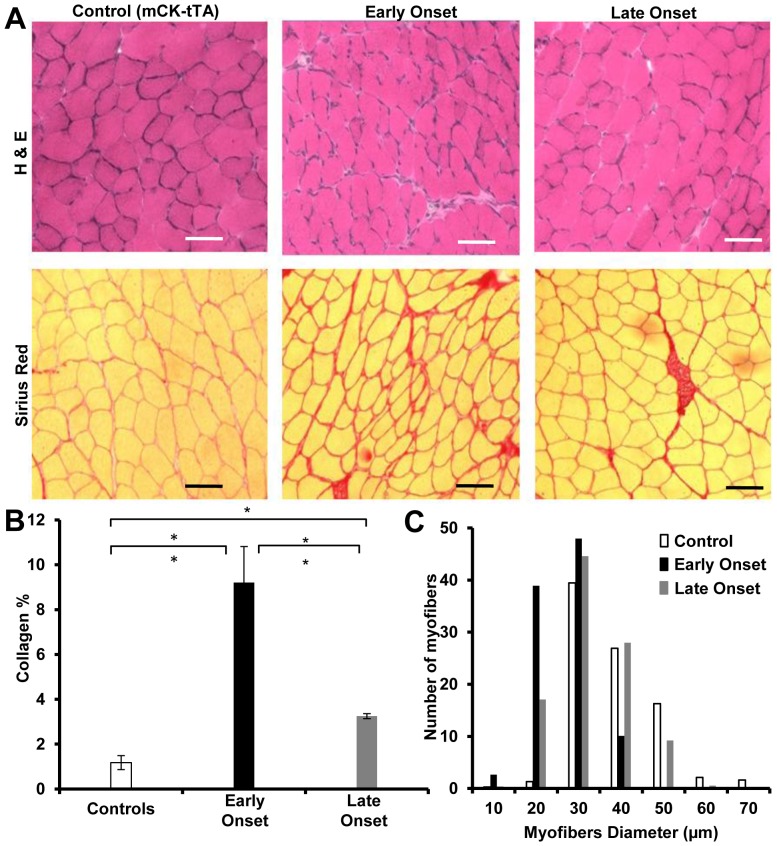
TGF-β1 over-expression leads to myofiber atrophy and endomysial fibrosis. (A) H&E and Pico-Sirius red staining showed smaller myofibers and collagen accumulation in the mice over-expressing TGF-β1. The differences were less prominent in the mice that did not develop muscle weakness at the time of muscle collection. Scale bar: 50 µm (B) Collagen deposition measurement after 2 weeks of TGF-β1 over-expression. The mice in the EO group showed significantly higher collagen deposition in comparison to LO and control mice. “*” indicates p<0.05 and “**” indicates p<0.01. (C) Fiber size distribution after 2 weeks of TGF-β1 over-expression. The myofiber size was reduced in both the EO and LO groups with more reduction in the EO group.

We performed H&E and Pico-Sirius red staining to examine muscle pathology and fibrosis accumulation, respectively in response to TGF-β1 expression. Quadriceps of six pairs of littermate with either EO or LO group and 4 control mice were examined after the TGF-β1 was over-expressed for 2 weeks. The H&E staining showed that no obvious muscle regeneration, degeneration or inflammation was induced in the muscles of the mice over-expressing TGF-β1. However, the mice developed excessive collagen deposition and the myofibers were smaller than the control mice (Figure 3A). To quantify the atrophy of myofibers observed in the muscles of the transgenic mice, we measured muscle fiber diameters using minimal Feret’s diameter measurement [26]. Myofiber diameters shifted toward smaller in size in the EO group (Figure 3 C). In addition, the mean myofiber diameters of the mice in EO group was significantly smaller (22.17±1.12 µm, p<0.005) compared to those of the LO group (28.23±2.6 µm) and the control mice (34.89±3.3 µm). The myofibers of the LO group were also significantly smaller (p<0.05) than the control mice. Collagen accumulation in the mice of EO group was 2.9 folds higher than the mice in the LO group (p<0.005) and 7.9 folds higher than the control mice (p<0.001). The collagen deposition in the LO group was significantly higher (p<0.05) than the control mice.

### Endogenous TGF-β1 was Induced in the *mCK-tTA/TRE-TGF-β1* Mice that Developed Phenotypes within 2 Weeks

**Figure 4:**
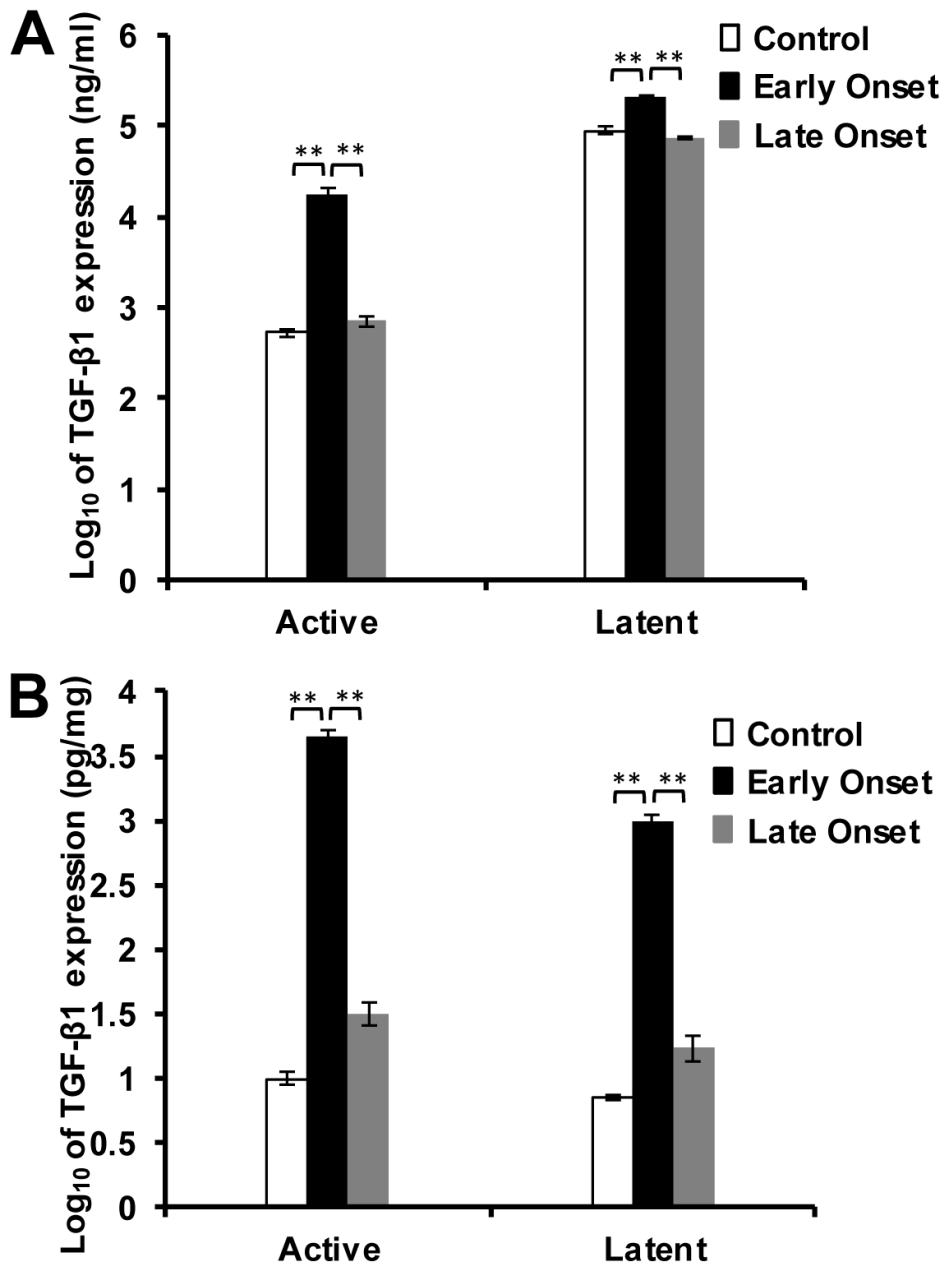
Active and latent TGF-β1 levels in serum and quadriceps after two weeks of *TGF-β1* over-expression. (A) The serum level of both active and latent TGF-β1 was significantly higher in mice in the EO group in comparison to LO and control littermates. (B) The level of both active and latent TGF-β1 in quadriceps was significantly higher in mice in the EO group in comparison to LO and control littermates. “*” indicates p<0.05 and “**” indicates p<0.01.

To determine whether the disease severity is associated with the expression level of the TGF-β1, both latent and active TGF-β1 in serum and muscle of EO, LO and control groups were measured by ELISA. To distinguish transgenic porcine TGF-β1 from endogenous mouse TGF-β1 we compared level of active and latent TGF-β1 since the transgene produces predominantly active TGF-β1. Since the antibody does not distinguish between the porcine TGF-β1 from the transgene and the endogenous murine TGF-β1, the measurement of the active TGF-β1 is a mixture of both. However, the measurements of the latent TGF-β1 indicate only the endogenous murine TGF-β1. In mice of the EO group both active and latent TGF-β1 in blood serum were significantly (p<0.01) elevated compared to those in the LO’s group (Table 1 and Figure 4A). In addition, both active and latent TGF-β1 in serum were also significantly (p<0.01) elevated in the mice of the EO group compared to those in the control mice (Table 1 and Figure 4A). The mice in the LO group did not show a significant difference in either active or latent forms of TGF-β1 in comparison to the control mice (Table 1and Figure 4A). Both active and latent TGF-β1 in the muscle lysates were significantly (p<0.01) elevated in the mice of the EO group compared to those in the control or LO groups (Table 1 and Figure 4B). The active and latent forms of TGF-β1 were not significantly different in the mice of the LO group compared to the control mice (Table 1 and Figure 4B).

**Table 1:** TGF-β1 protein levels in the blood serum and muscle lysates.

Mice	Serum (mean ± s.e.m., ng/ml)		Muscle (mean ± s.e.m., pg/mg of muscle)	
	Active	Latent	Active	Latent
Early Onset (EO)	23.27±5.58	217.34±18.87	5243.25±1275.92	1194.33±321.43
Late Onset (LO)	0.83±0.22	75.28±5.97	91.10±57.81	42.52±31.28
Control	0.55±0.10	94.72±19.48	22.43±6.92	7.24±0.57

A previous study showed that genetic polymorphism involving a 36-bp deletion within exon 12 in the latent transforming growth factor binding protein-4 (*Ltbp4*) gene was able to affect the activation of TGF-β1 [20]. The shorter allele with a 36-bp deletion encodes an LTBP4 protein that enhances the release of active TGF-β1 by increasing the proteolysis of the proline-rich region of LTBP4 [20]. To determine whether this polymorphism contributes to the phenotypic variations observed in the TGF-β1 transgenic mice, we examined *Ltbp4* genotypes in these mice. The PCR results showed no association between this genetic polymorphism and the EO/LO phenotypes (Figure S1).

## Discussion

Increased levels of TGF-β1 have been demonstrated in DMD, X-linked spinal and bulbar muscular atrophy, congenital muscular dystrophies and inflammatory myositis patients [5], [22], [23], [27], [28], [29]. TGF-β1 is believed to play a critical role in the fibrosis observed in the affected muscles and may contribute to the failure of regeneration in some of the diseases such as DMD. TGF-β1 promotes growth and differentiation of fibroblasts and is a potent regulator of collagen synthesis [30], [31]. In addition, TGF-β1 can convert satellite cells into myofibroblasts, which promotes fibrosis and potentially depletes the satellite cells for muscle regeneration [16], [30]. In addition to promoting fibrosis, TGF-β1 can suppress myoblasts differentiation in culture [32]. Local delivery of TGF-β1 by single intramuscular or subcutaneous injection into the hindlimbs of mice leads to muscle atrophy and fibrosis phenotypes [16], [33]. In one study, intramuscular injection of 5 ng of TGF-β1 lead to inflammatory responses within 7 days, which disappeared at a later time point. However, the inflammation was not observed in a second study in which 0.2 ng of TGF-β1 was delivered by subcutaneously injection [16], [33]. In our study, we observed only endomysial fibrosis and muscle atrophy in the *mCK-tTA/TRE-TGF-β1* mouse model after the transgene was over-expressed, without cellular infiltration observed in muscles, suggesting that inflammation occurred in the first study might be induced by a combination of the presence of TGF-β1 and muscle injuries caused by the intramuscular injections.

Our study showed 22.43 pg of TGF-β1 per mg total protein in skeletal muscles of control mice. Endomysial fibrosis and myofiber atrophy were detected in the LO group of mice, which showed 4 fold-up-regulation of TGF-β1. No significant muscle weakness was detected in these mice. In the EO group, an average of 234 fold up-regulation of active TGF-β1 lead to pathological changes, muscle weight loss and muscle weakness. Our data suggest a dose related response to TGF-β1 *in vivo*. When individual muscles were examined, we observed that the response to TGF-β1 over-expression was muscle-type-specific. The loss of muscle mass was most severe in diaphragm which has been shown to be more affected in DMD and its animal models [33], [34], [35], [36], [37], [38].

While the muscle fibrosis and myofibers atrophy are prominent pathological changes in the mice over-expressing TGF-β1, we did not observed degeneration/regeneration or overt inflammatory responses in the muscles of these mice. TGF-β1 is a pleiotrophic cytokine, which has both inflammatory and anti-inflammatory effects [39]. Complete elimination of TGF-β1 in TGF-β1^−/−^ mice lead to intrauterine or premature death and excessive inflammatory responses in multiple tissues [40]. Suppression of TGF-β1 using neutralizing antibodies in *mdx* mice has been reported to either increase skeletal muscle inflammation or have no obvious effect [24], [41]. Our data showed that the severe endomysial fibrosis induced by high level of TGF-β1 is independent of inflammation in the muscle.

The time of onset was various after the *TGF-β1* transgene was induced in the *mCK-tTA/TRE-TGF-β1* mice. Enzyme-linked immunosorbent assays (ELISA) showed that the time of the onset correlate to the amount of TGF-β1 in both the muscles and in circulation. Interestingly, the protein level of the latent TGF-β1 in the muscles was up-regulated in both EO and LO groups with a significant difference between the two groups, indicating that the endogenous TGF-β1 had been induced in the muscles with a higher level in the mice that showed more severe phenotypes. The findings suggest an autocrine regulation of TGF-β1 expression in skeletal muscles. Since both the active and latent TGF-β1 were also found up-regulated in sera, we cannot rule out the possibility that other cell types such as fibroblasts and immune cells are involved in the positive feedback loop as previously reported in pancreatic stellate cells [42]. In this study we crossed two transgenic lines to generate the *mCK-tTA/TRE-TGF-β1* mice, therefore the mice likely to carry more polymorphisms in their genomes than pure breed lines. A polymorphism in *Ltbp4* gene has been shown to modulate TGF-β1 activation [20]. To determine whether this polymorphism contributes to the phenotypic variations observed in the TGF-β1 transgenic mice, we examined *Ltbp4* genotypes in these mice. Our results showed that this polymorphism did not contribute to the phenotypic variations observed (Figure S1). Additional investigations are needed to identify genes that modify the TGF-β1 activities in the mice.

A recent genetic study showed that a polymorphism in the promoter of osteopontin (OPN), which reduces the expression level of OPN, was associated with the more severe disease phenotype in DMD [43]. Like TGF-β1, OPN has been associated with fibrosis formation in different diseases [44], [45], [46]. The *mdx* mice lacking OPN gene developed milder phenotype with significant reduction of fibrosis [45]. The study conducted in *mdx* mice suggested that OPN directly modulate TGF-β1 signaling [45]. Interestingly, after two weeks of TGF-β1 expression, expression of OPN is strongly induced in the *mCK-tTA/TRE-TGF-β1* mice (unpublished microarray data). In addition, the expression significantly correlated with the expression of TGF-β1 (r^2^ = 0.088), suggesting OPN may be part of the autocrine feedback loop and modify the expression of TGF-β1.

While most studies reported negative regulatory role of TGF-β1 in myogenesis, others showed that TGF-β1 plays an essential role in muscle regeneration [47], [48], [49]. This conditional muscle-specific mouse model provides a useful tool for examining the regulatory roles of TGF-β1 in skeletal muscles and for developing strategies to modulate TGF-β1 in muscles.

## Materials and Methods

### Generation of Tet-repressible Muscle Specific Transgenic *TGF-β1* Mice

All animal procedures were approved by the institutional animal care and use committee (IACUC) at the Children’s Research Institute. Two transgenic mouse lines (*TRE-TGF-β1* and *mCK-tTA*) were cross-bred in order to generate the tet-repressible muscle-specific TGF-β1 transgenic mice (*TRE-TGF-β1/mCK-tTA*). The *TRE-TGF-β1* line carries a porcine *TGF-β1* cDNA containing a double mutation where cysteines at positions 223 and 225 are converted to serines, which is regulated by the tet*O* recognition element (TRE) [50]. The *mCK-tTA* line carries a construct containing the tetracycline-controlled transactivator (tTA) protein driven by a muscle specific creatine kinase promoter (*mCK*) [20], [51]. The presence of doxycycline in cells inhibits binding of tTA to the TRE and blocks *TGF-β1* transgene expression (Figure S2). After cross-breeding the *TRE-TGF-β1* and *mCK-tTA* lines, the pregnant female mice received drinking water with doxycycline (200 µg/ml in 5.0% sucrose) in order to suppress the *TGF-β1* transgene expression in the pups *in utero*. Under these conditions, the *TRE-TGF-β1/mCK-tTA* transgenic mice were born at an expected Mendelian ratio. After weaning, all pups were maintained on water treated with doxycycline until they were entered into an experimental regiment. In this study, doxycycline was removed from water to induce transgene expression in the *TRE-TGF-β1/mCK-tTA* mice along with control when the mice were between 4 to 14 weeks old. For all experiments, littermates carrying single transgene *tTA* were used as controls. Genotyping primers used for polymerase chain reaction (PCR) were: TGF-β1, forward, 5′-GGGCTACCATGCCAATTTCTG–3′; reverse, 5′-GTACAGAGCCAGGACCTTGCTG-3′. tTA, forward, 5′-ACAGCGCATTAGAGCTGCTT-3′; reverse, 5′-CCCCTTCTAAAGGGCAAAAG-3′. The PCR amplicons were visualized using 2% agarose gels (Gibco BRL, Gaithersburg, MD). The *TGF-β1* primers target a 5′UTR region that is specific to the transgene therefore does not amplify murine *Tgf-β1* gene. The age of mice when TGF-β1 was induced and assays performed for phenotype characterizations are summarized in table S1.

### Body and Muscle Mass

The TGF-β1 expression was induced in the muscles of twenty 7–14 weeks old *TRE-TGF-β1/mCK-tTA* mice by discontinuing oral doxycycline. Thirteen female and seven male *TRE-TGF-β1/mCK-tTA* mice, and their age- and gender-matched single transgenic littermates were studied. The body weight of the mice was measured 3 times a week starting from the date that the doxycycline was stopped. Onset of the disease phenotype was determined by weight loss and muscle strength measured by grip strength test. The mice were euthanized for muscle collection at the onset of the disease phenotype. Masseter, soleus, tibialis anterior, quadriceps, gastrocnemius, deltoid, triceps, biceps brachii and the diaphragm from three 12 weeks old female *TRE-TGF-β1/mCK-tTA* transgenic mice and three control littermates were dissected and immediately weighed to determine the muscle weight changes.

### Muscle Grip Strength Test

The muscle grip strength test was performed as previously described [51]. The mice monitored for body weight changes were tested for muscle strength two weeks after the TGF-β1 induction. Briefly, the grip strength meter (GSM) (Columbus Instruments, Columbus, OH) consists of two steel grids connected to force meters. Grip strength is tested by holding the mouse over a grid of GSM until the mouse can grip the steel bars. Then the mouse is pulled away from the force meter until it releases the grid. The meter recorded the maximum force that was applied. The mice were acclimated to the GSM for five minutes one day prior to data collection. They were then tested once a day for five consecutive days. Five measurements were recorded for each test. The highest values of the five tests each day were averaged and normalized to body weight (kg).

### Pico-Sirius Red and H&E Staining

TGF-β1 over-expression was induced when the mice were 4 weeks old then their muscles were collected after 2 weeks. Muscles of six mice that showed body weight lost and muscle weakness within two weeks (EP) and 6 littermates that did not show the phenotype within 2 weeks (LP) were examined for pathological changes, changes of myofiber sizes and collagen accumulation. Please note that the mice in the EO and LO groups were collected in pairs from the same litters. Immediately after dissection, the muscles were snap-frozen in isopentane cooled with liquid nitrogen, then stored at −80°C. A Leica CM 1900 cryostat (Walldorf, Baden-Wurttenberg, Germany) was used to prepare cryosections for the pathohistological analysis. Hematoxylin and Eosin (H&E) staining was conducted using 8 µm sections as previously described [51]. Five random non-overlapping fields of the tissue section (40X) were imaged using Nikon Eclipse E800 microscope (Nikon, Chiyoda-ku, Tokyo, Japan), RT slider camera (Diagnostic Instrument, Sterling Height, MI) and SPOT advanced software. The diameters of myofibers were determined by measuring the minimal Feret’s diameter using the SPOT Advanced software as previously described [51].

Pico-Sirius Red staining was performed to measure accumulated collagen in the gastrocnemius muscles. Nuclei of 8 µm muscle sections were stained with Weigert’s Hematoxyline (Sigma Aldrich, St. Louis, MO) for 5 minutes followed by Pico-Sirius red (Sigma Aldrich, St. Louis, MO) staining for 1 hour. The sections were washed using acidified water with 0.5% glacial acetic acid (Sigma Aldrich, St. Louis, MO), dehydrated in 100% ethanol then submerged in xylene. The digital images were processed using Image J (http://rsb.info.nih.gov/ij). Pixels corresponding to the area stained in red were normalized to the total pixel area of the tissue and the results were expressed as percentage of collagen accumulations [52].

### Enzyme-linked Immunosorbent Assay (ELISA)

The amount of active TGF-β1 and total TGF-β1 (active and latent) in skeletal muscles and sera was determined using TGF-β1 immunoassay system (R&D systems, Minneapolis, MN). The sera were collected from the same 6 pairs of mice used for pathological examinations. The sera were diluted 10 times and the active TGF-β1 was determined using the ELISA plate pre-coated with TGF-β1 specific antibody following the manufacture’s protocol. To determine the amount of latent TGF-β1, the sera were treated with 1N HCl to activate the latent TGF-β1 before measurement using the ELISA kit. The amount of the latent TGF-β1 was then be calculated by subtracting the amount of the active TGF-β1 from the total amount of TGF-β1 determined. The optical density was determined at 450 nm within 30 minutes after the reactions were stopped. The wavelength correction was performed by subtracting the value at 570 nm from that at 450 nm. The amount of the active and latent TGF-β1 in muscles was determined using muscles lysate. Quadriceps muscles were homogenized in RIPA buffer prior to ELISA using the ELISA kit (R&D systems, Minneapolis, MN).

### *Ltbp 4* Genotyping by Polymerase Chain Reaction (PCR)

We performed PCR to examine a polymorphism in *Ltbp4* gene that was reported to be associated with TGF-β1 activation [20]. The primers were: forward, 5′-AGAACCCTGGACAGATG–3′; reverse, 5′-TCCAAGCAGGGATGC-3′. A 260 bp PCR amplicon represents the long allele and a 224 bp PCR amplicon represents the short allele (36-bp deletion) were visualized using 2% agarose gels (Figure S1).

## Supporting Information

Figure S1 **No association between** ***Ltbp 4*** **genotypes and the phenotypes of** ***mCK-tTA*** **/** ***TRE-TGF-β1*** **mice.** The long allele (260 bp) and short allele (224 bp) were not associated with the early (EO, lane 1, 4, 6) and late (LO, lane 2, 3, 5) phenotypes. Lane 7 and 8 show *Ltbp 4* genotypes of 2 control mice.
(TIF)

Figure S2 **Tet-repressible system of the** ***mCK-tTA*** **/** ***TRE-TGF-β1*** **mice.** The expression of tetracycline-controlled transactivator (tTA) is regulated by the promoter of muscle creatine kinase (*mCK*). The TGF-β1 transgene expression is regulated by tetracycline-response element (TRE) which consists of a heptameric tetO sequence and a minimal CMV promoter (*P*minCMV). In the presence of doxycycline, the doxycycline will interact with the tTA, which prevents the tTA bind to the TRE. In the absence of doxycycline, the tTA binds the TRE, which activates transcription of the *TGF-β1* transgene.
(TIF)

Table S1 **The age of mice when TGF-β1 was induced and assays performed for phenotype characterizations.**
(DOC)
