# Supplementary figures and images for: Conditional Expression of TGF-β1 in Skeletal Muscles Causes Endomysial Fibrosis and Myofibers Atrophy
Source: PLoS One. 2013 Nov 14;8(11):e79356. doi: 10.1371/journal.pone.0079356 (PMC3828351; doi:10.1371/journal.pone.0079356)

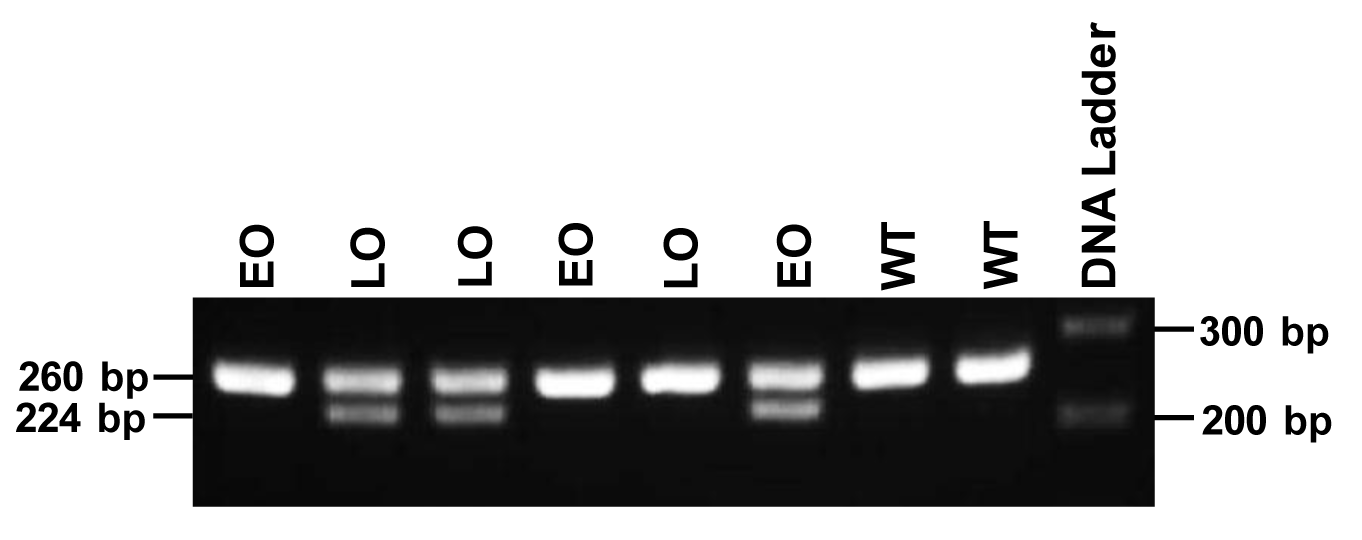

Supplement: Figure S1 — No association between Ltbp 4 genotypes and the phenotypes of mCK-tTA / TRE-TGF-β1 mice. The long allele (260 bp) and short allele (224 bp) were not associated with the early (EO, lane 1, 4, 6) and late (LO, lane 2, 3, 5) phenotypes. Lane 7 and 8 show Ltbp 4 genotypes of 2 control mice. (TIF) [file pone.0079356.s001.tif]

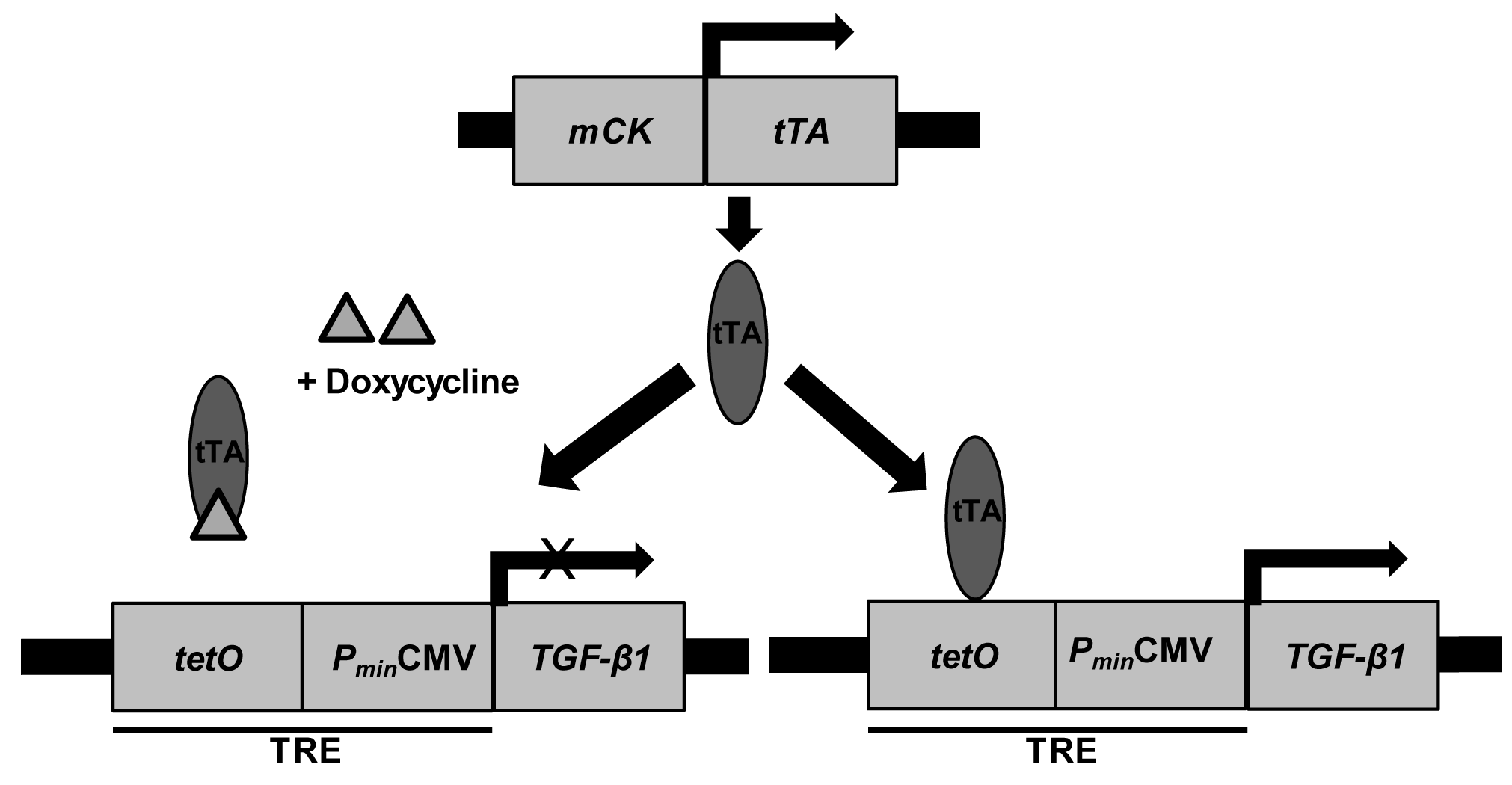

Supplement: Figure S2 — Tet-repressible system of the mCK-tTA / TRE-TGF-β1 mice. The expression of tetracycline-controlled transactivator (tTA) is regulated by the promoter of muscle creatine kinase (mCK). The TGF-β1 transgene expression is regulated by tetracycline-response element (TRE) which consists of a heptameric tetO sequence and a minimal CMV promoter (PminCMV). In the presence of doxycycline, the doxycycline will interact with the tTA, which prevents the tTA bind to the TRE. In the absence of doxycycline, the tTA binds the TRE, which activates transcription of the TGF-β1 transgene. (TIF) [file pone.0079356.s002.tif]
