# Supplementary material for: Conditional Expression of TGF-β1 in Skeletal Muscles Causes Endomysial Fibrosis and Myofibers Atrophy
Source: PLoS One. 2013 Nov 14;8(11):e79356. doi: 10.1371/journal.pone.0079356 (PMC3828351; doi:10.1371/journal.pone.0079356)
Supplement: Table S1 — The age of mice when TGF-β1 was induced and assays performed for phenotype characterizations. (DOC) [file pone.0079356.s003.doc]

| Sample size (n) | TGF-β1 induction  (age in weeks) | Assays performed  (age in weeks) |
| --- | --- | --- |
| 20 | 7-14 weeks old | Body weight measurement 3 times a week; grip strength measurement every other week. |
| EO:10  LO:11  Control: 8 | 4 weeks old | Grip strength measurement (6 weeks old) |
| EO: 6  LO: 6  Control : 4 | 4 weeks old | H&E, collagen staining, fiber diameter, *Ltbp4* genotyping and ELISA (6 weeks old) |
